# Fructose Beverage Consumption Induces a Metabolic Syndrome Phenotype in the Rat: A Systematic Review and Meta-Analysis

**DOI:** 10.3390/nu8090577

**Published:** 2016-09-20

**Authors:** Carla R. Toop, Sheridan Gentili

**Affiliations:** School of Pharmacy and Medical Sciences, Sansom Institute for Health Research, University of South Australia, Adelaide 5000, SA, Australia; carla.toop@mymail.unisa.edu.au

**Keywords:** fructose, beverage, rat, metabolic syndrome, meta-analysis, diabetes, obesity

## Abstract

A high intake of refined carbohydrates, particularly the monosaccharide fructose, has been attributed to the growing epidemics of obesity and type-2 diabetes. Animal studies have helped elucidate the metabolic effects of dietary fructose, however, variations in study design make it difficult to draw conclusions. The aim of this study was to review the effects of fructose beverage consumption on body weight, systolic blood pressure and blood glucose, insulin and triglyceride concentrations in validated rat models. We searched Ovid Embase Classic + EmbaseMedline and Ovid Medline databases and included studies that used adolescent/adult male rats, with fructose beverage consumption for >3 weeks. Data from 26 studies were pooled by an inverse variance weighting method using random effects models, expressed as standardized mean differences (SMD) with 95% confidence intervals (CI). Overall, 10%–21% *w*/*v* fructose beverage consumption was associated with increased rodent body weight (SMD, 0.62 (95% CI: 0.18, 1.06)), systolic blood pressure (SMD, 2.94 (95% CI: 2.10, 3.77)) and blood glucose (SMD, 0.77 (95% CI: 0.36, 1.19)), insulin (SMD, 2.32 (95% CI: 1.57, 3.07)) and triglyceride (SMD, 1.87 (95% CI: 1.39, 2.34)) concentrations. Therefore, the consumption of a low concentration fructose beverage is sufficient to cause early signs of the metabolic syndrome in adult rats.

## 1. Introduction

A high intake of refined carbohydrates and sweeteners, including sucrose and high fructose corn syrup (HFCS), have been attributed to the growing epidemics of obesity and type-2 diabetes (T2D) in Western society [1]. Current evidence suggests that the consumption of added dietary sugars (including sucrose and HFCS) is currently stable or decreasing [2], however, it still remains high. It is estimated that Australians consume approximately 46.83 kg of sugar per year, while Americans consume on average 68.57 kg per year [3]. This translates to between 26.8% and 39.3% of daily energy consumed from added sugar (based on 2000 Cal/day). Furthermore, sugar consumption is high in both children and adolescents [4], which has been identified as contributing to the alarming rates of T2D and obesity observed in this population [5]. Specifically, the monosaccharide fructose has been identified as a key component of added sugars contributing to these epidemics. Despite the evidence, there is still confusion surrounding whether the consumption of fructose at physiologically relevant concentrations contributes to the development of metabolic disease. This is not surprising due to the varying outcomes found by studies investigating the effects of fructose consumption on human metabolic health [6,7,8].

While the western diet is typically low in free fructose, the main dietary sources of fructose are sucrose, HFCS, fruits and honey. Despite having the same chemical formula as glucose, hepatic fructose metabolism is different [9]. In the rodent, chronic consumption of fructose at high concentrations is known to give rise to ectopic fat deposition, insulin resistance, T2D and elevated blood pressure [9]. The evidence associated with lower concentration fructose beverage consumption, however, is less clear [6].

Fructose feeding is an established experimental model for inducing the metabolic syndrome in rats [10,11]; however, studies vary significantly in fructose delivery and the administered concentration. The concentration of fructose administered as a component of chow itself is generally supraphysiological and ranges between 60%–70% *w*/*w* or 0.6–0.7 g/g chow [10,12,13,14,15,16,17,18,19,20,21], whereas the concentration of fructose beverages can often vary anywhere between 10%–30% *w*/*v* or 0.1–0.3 g fructose/mL water [22,23,24,25,26,27,28,29,30,31,32,33,34,35,36,37,38,39,40,41,42,43,44,45]. Despite the reported effects on metabolic health, studies investigating the effect of high concentrations of fructose in chow or as a beverage report no effect of fructose consumption on rodent body weight [10,12,46,47], while others report an increase in body weight [22,23,27,29,30,38,39,40,41,43,48,49]. Furthermore, while these studies give an understanding of the effects associated with excess fructose intake, the physiological outcomes associated with supraphysiological concentrations of fructose cannot be used to extrapolate the effects to human health. Thus, the effect of lower concentration fructose beverage consumption at concentrations similar to those found in sugar-sweetened beverages (~10% *w*/*v*) must be clarified in a rodent model.

To provide consistent evidence of the metabolic effects of fructose, we undertook a systematic review and meta-analysis of experimental animal studies to assess the effect of low concentration fructose beverage administration on rodent body weight, systolic blood pressure and blood glucose, insulin and triglyceride concentrations. To our knowledge, this is the first meta-analysis to explore the effects of administration of a fructose beverage in a rodent model.

## 2. Materials and Methods

### 2.1. Literature Search

**Figure 1:**
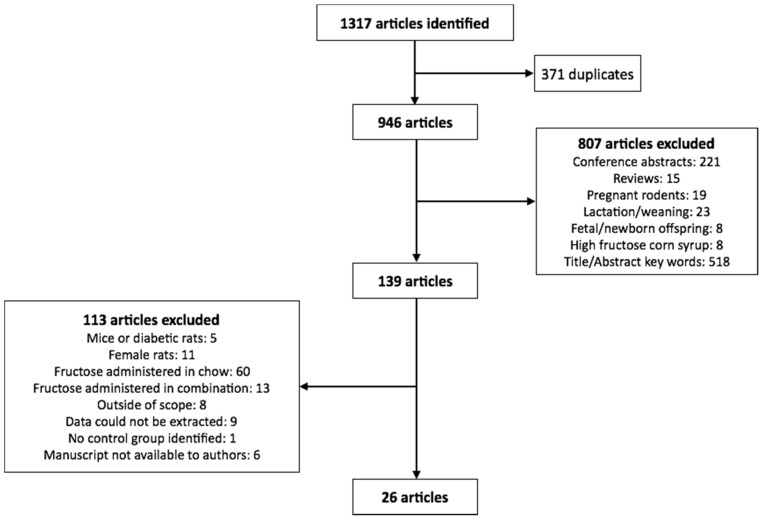
Flowchart of studies selected for the meta-analysis.

We searched Ovid Embase Classic + EmbaseMedline and Ovid Medline for articles published within the last 10 years with medical subject headings (MeSH) terms fructose, rat or rats, and weight. Only articles published in English were included in the study. From this, 1317 eligible articles were identified, of which 371 were identified as duplicates. The remaining 946 articles were reviewed for inclusion in the analysis. The exclusion search criteria included: conference abstracts, review articles, studies utilizing pregnant rodents, studies completed on rodents during the lactation or weaning periods, studies conducted during rodent development (i.e., fetuses or offspring less than 8 weeks of age), HFCS feeding, and citations which did not list fructose or body weight in the title or abstract (Figure 1).

### 2.2. Study Selection

Of the remaining 139 citations, only studies which used male adolescent/adult rodents (>8 weeks of age) were included in the analysis, in which fructose alone (not in combination with glucose or as sucrose or HFCS) was administered in a beverage where the concentration was provided. Of these, studies that included numerical data (mean ± standard deviation) on at least two of; final body weight, systolic blood pressure and blood glucose, insulin and triglyceride concentrations were included (26 studies in total as summarized in Figure 1 and Table 1).

### 2.3. Data Collected

We collected the mean and standard deviation data for body weight (g), systolic blood pressure (mmHg), blood glucose (mmol/L), blood insulin (pmol/L) and blood triglyceride (mmol/L) concentrations in animals exposed to control and fructose as reported in the publications. Where required, the glucose, insulin and triglyceride concentrations were converted to the above listed units. Where reported, the major findings of fructose on each biological measure is summarized in Table 1.

### 2.4. Statistical Analysis

Data were expressed as standardized mean differences (SMD) with 95% confidence intervals (CI). Heterogeneity was assessed with the *Q* and *I*^2^ statistics. *Q* values *p* < 0.10 and *I*^2^ ≥ 85% were taken to indicate heterogeneity [51]. For all analyses, a random-effects model was used due to the significant heterogeneity associated with study design, rodent strain (not assessed as part of this analysis) and the duration of intervention. Where possible, the effects of study duration (fructose consumption for ≤12 weeks versus >12 weeks) on fructose-induced changes in body weight, systolic blood pressure and blood glucose, insulin and triglyceride concentrations were determined. All analyses were completed in R-Studio using the meta package (R-Studio version 0.99.491: Integrated Development for R. RStudio, Inc., Boston, MA, USA; meta package version 4.3-2) [52].

## 3. Results

### 3.1. Study Characteristics

This analysis only included studies in which fructose was administered as a beverage to adolescent or adult male rodents. Fructose was supplied at a concentration of either 10% *w*/*v* (24 studies) or 20%–21% *w*/*v* (two studies), and was administered for a period ranging from 2.9 to 38 weeks (Table 1). A seminal study by Hwang et al. showed that the metabolic syndrome can be induced after only 2 weeks of fructose consumption at a concentration of 60% of daily calories [10]. However, consumption of fructose at such supraphysiological concentrations is rare in humans. When planning this study our intention was to group all studies, independent of study duration. However, whilst collating the data we noted divergent study durations of either less than or greater than 12 weeks (refer to summary statistics in Table 2). We found no difference in the concentration of fructose administered or the sample size between the two groups, therefore data were split for all subsequent analyses. Of the 26 studies identified, 20 reported raw data on final body weight following fructose consumption. Of these, 35.0% reported that fructose consumption significantly increased rodent body weight at the end of the study period (Table 1). Similarly, of the 23 studies that reported blood glucose concentration, only 30.4% reported a significant effect of fructose consumption. Significant increases in blood insulin, blood triglycerides and systolic blood pressure were reported in 84.2%, 87.5% and 100%, respectively, for studies in which this data were reported (Table 1).

### 3.2. Effect of Fructose on Rodent Body Weight and Systolic Blood Pressure

**Figure 2:**
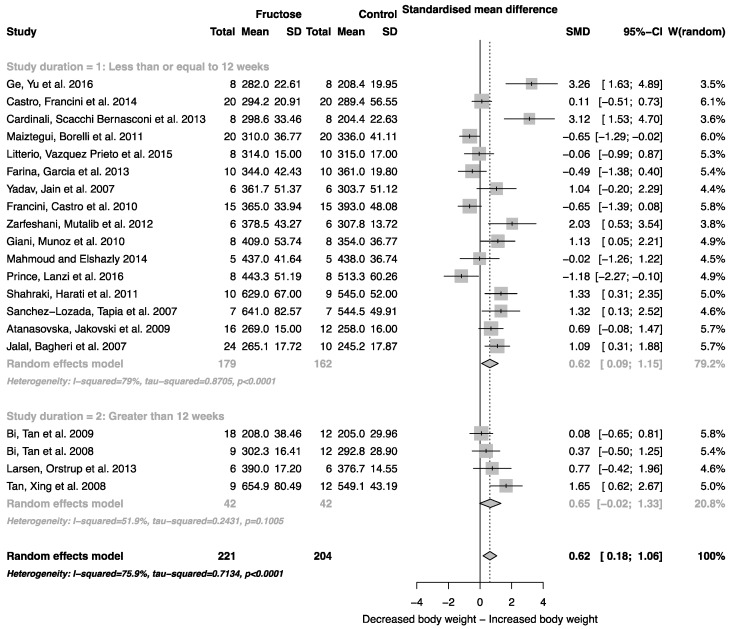
Forest plots of the effect of fructose consumption on adult male rodent body weight (mean and standard deviation (SD), split by study duration. The pooled effects estimates are represented by three diamonds; one for studies of 12 weeks or less, one for studies of greater than 12 weeks, and one representing the combined effect. Data are presented as standardized mean differences (SMD) with 95% confidence interval (CI). *p*-Values are for the inverse variance random effects models with DerSimonian-Laird estimator for Tau^2^. Inter-study heterogeneity was tested by Cochran’s *Q* at a significance of *p* < 0.10 and quantified by *I*^2^.

When all studies that reported mean body weight and standard deviation were combined, there was an overall effect of fructose consumption on rodent body weight (Figure 2; SMD, 0.62 (95% CI: 0.18, 1.06); *z* = 2.79; *p* = 0.005). It is important to note, however, the high degree of heterogeneity across these studies (*I*^2^ = 75.9% (95% CI: 62.9, 84.3)). There was no effect of study duration (between group difference *p* = 0.944) on rodent body weight.

**Figure 3:**
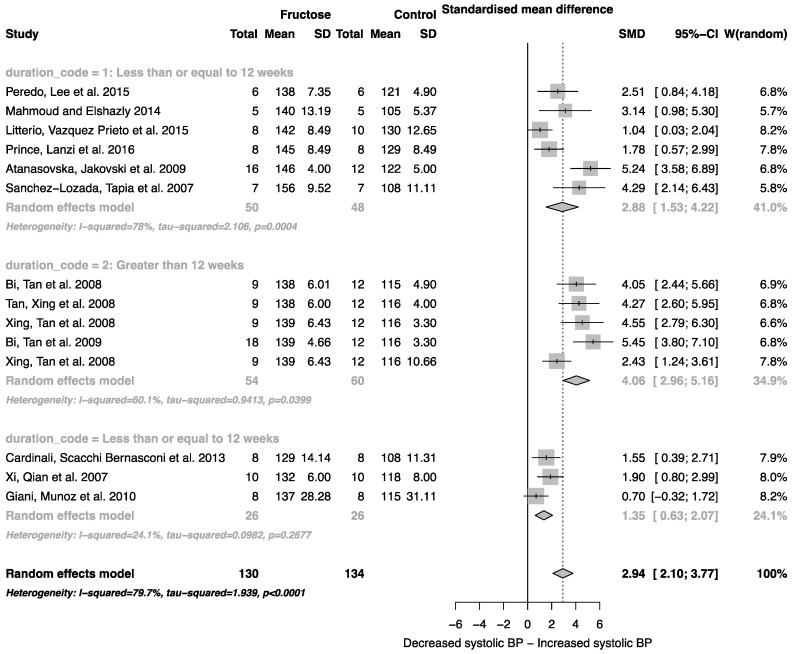
Forest plots of the effect of fructose consumption on adult male systolic blood pressure (mean and standard deviation (SD)), split by study duration. The pooled effects estimates are represented by three diamonds; one for studies of 12 weeks or less, one for studies of greater than 12 weeks, and one representing the combined effect. Data are presented as standardized mean differences (SMD) with 95% confidence interval (CI). *p*-Values are for the inverse variance random effects models with DerSimonian-Laird estimator for Tau^2^. Inter-study heterogeneity was tested by Cochran’s *Q* at a significance of *p* < 0.10 and quantified by *I*^2^.

Fructose consumption was associated with a strong effect on systolic blood pressure (SMD, 2.94 (95% CI: 2.10, 3.77); *z* = 6.91, *p* < 0.0001; Figure 3). Furthermore, there was a significant effect of study duration on systolic blood pressure (between group difference *p* = 0.0002), with fructose consumption for greater than 12 weeks resulting in a significant increase in systolic blood pressure when compared to less than or equal to 12 weeks (Figure 3).

### 3.3. Effect of Fructose on Rodent Blood Glucose, Insulin and Triglyceride Concentrations

**Figure 4:**
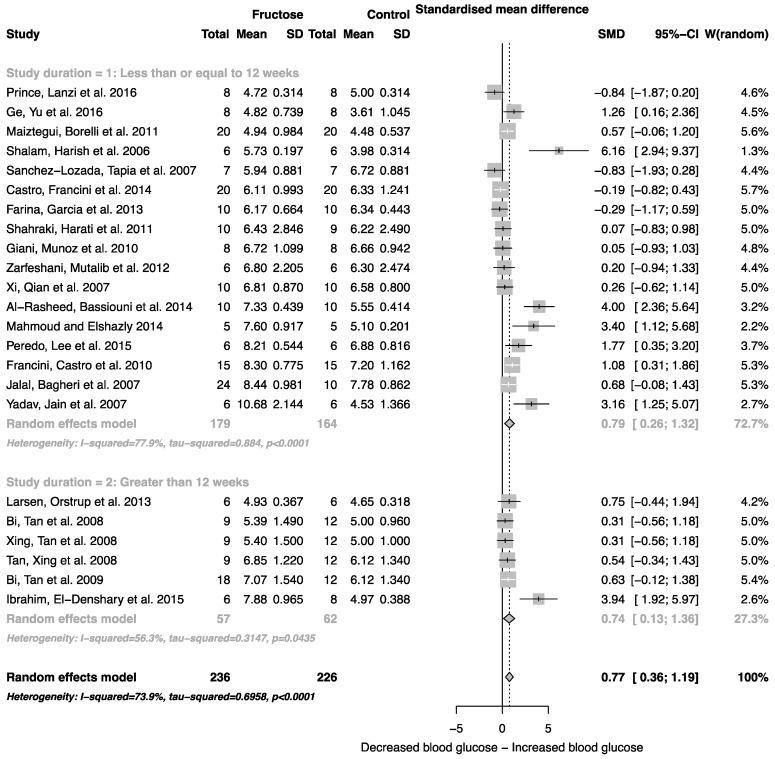
Forest plots of the effect of fructose consumption on adult male rodent blood glucose concentration (mean and standard deviation (SD)), split by study duration. The pooled effects estimates are represented by three diamonds; one for studies of 12 weeks or less, one for studies of greater than 12 weeks, and one representing the combined effect. Data are presented as standardized mean differences (SMD) with 95% confidence interval (CI). *p*-Values are for the inverse variance random effects models with DerSimonian-Laird estimator for Tau^2^. Inter-study heterogeneity was tested by Cochran’s *Q* at a significance of *p* < 0.10 and quantified by *I*^2^.

**Figure 5:**
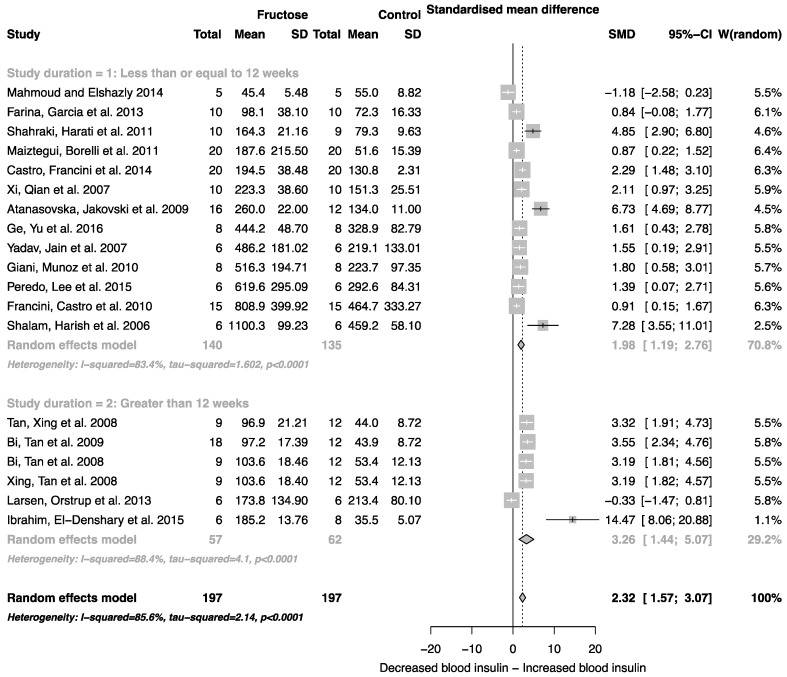
Forest plots of the effect of fructose consumption on adult male rodent blood insulin concentration (mean and standard deviation (SD)), split by study duration. The pooled effects estimates are represented by three diamonds; one for studies of 12 weeks or less, one for studies of greater than 12 weeks, and one representing the combined effect. Data are presented as standardized mean differences (SMD) with 95% confidence interval (CI). *p*-Values are for the inverse variance random effects models with DerSimonian-Laird estimator for Tau^2^. Inter-study heterogeneity was tested by Cochran’s *Q* at a significance of *p* < 0.10 and quantified by *I*^2^.

**Figure 6:**
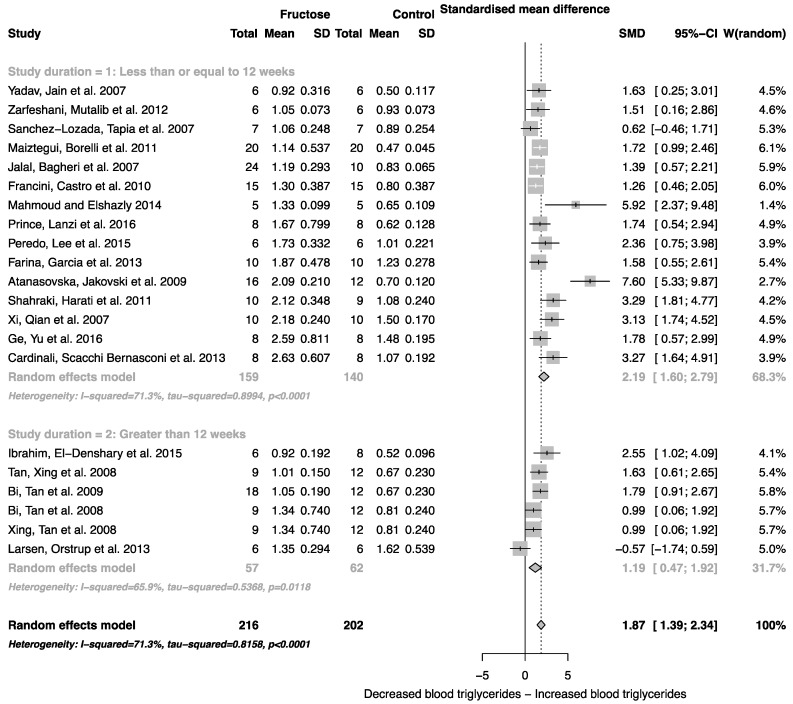
Forest plots of the effect of fructose consumption on adult male rodent blood triglyceride concentration (mean and standard deviation (SD)), split for study duration. The pooled effects estimates are represented by three diamonds; one for studies of 12 weeks or less, one for studies of greater than 12 weeks, and one representing the combined effect. Data are presented as standardized mean differences (SMD) with 95% confidence interval (CI). *p*-Values are for the inverse variance random effects models with DerSimonian-Laird estimator for Tau^2^. Inter-study heterogeneity was tested by Cochran’s *Q* at a significance of *p* < 0.10 and quantified by *I*^2^.

The effect of fructose consumption on blood glucose, insulin and triglyceride concentrations is summarized in Figure 4, Figure 5 and Figure 6, respectively. Overall, there was an effect of fructose consumption on blood glucose (Figure 4; SMD, 0.77 (95% CI: 0.36, 1.19); *z* = 3.64, *p* = 0.003), insulin (Figure 5; SMD, 2.32 (95% CI: 1.57, 3.07); *z* = 6.09, *p* < 0.0001) and triglyceride (Figure 6; SMD, 1.87 (95% CI: 1.39, 2.34); *z* = 7.70, *p* < 0.0001) concentrations. Subgroup analysis showed no effect of study duration on blood glucose (between group difference *p* = 0.9332) or insulin (between group difference *p* = 0.2042). Interestingly, the subgroup analysis suggested an effect of study duration on blood triglyceride concentration (between group difference *p* = 0.037), with fructose beverage consumption for greater than 12 weeks resulting in a significant decrease in blood triglyceride concentration when compared to less than or equal to 12 weeks (Figure 6).

## 4. Discussion

Fructose is commonly used in experimental animal models to induce features of the metabolic syndrome [10,11,53], however, the physiological impact of fructose varies depending on the concentration administered and the route of administration. This variation in study design has led to inconsistencies in the published effects of fructose consumption, which has made it difficult to extrapolate and understand the impact that fructose may have on human health. This study has shown that low concentration fructose beverage consumption, independent of variations in study design and duration, results in an increase in rodent body weight, systolic blood pressure and blood glucose, insulin and triglyceride concentrations.

The meta-analysis presented confirms that, within the limits of the studies undertaken, fructose beverage consumption at concentrations consistent with sugar-sweetened beverages (~10% *w*/*v*), results in an increase in rodent body weight, independent of study duration. Furthermore, we have shown that fructose beverage consumption is associated with increased blood triglyceride concentration and, together, this may suggest that the increase in body weight reported may be due to increased adipose tissue mass. Fructose feeding in rodents is commonly associated with increased hepatic de novo lipogenesis, leading to increased plasma triglycerides [12,14], non-esterified fatty acids (NEFA) [54] and very low-density lipoprotein (VLDL) cholesterol [16,19]. Furthermore, it is associated with increased hepatic lipogenic gene expression [16,55]. Fructose-induced increases in hepatic fatty acid synthesis are associated with increased ectopic fat deposition in a number of peripheral tissues including the liver. This may contribute to the increase in liver weight reported in several studies [17,48], and acts to promote hepatic inflammation and oxidative stress [17,55]. Interestingly, only a few rodent studies have reported an overall increase in body adipose tissue mass following fructose consumption [48,56,57], despite its known lipogenic effects.

Elevated plasma uric acid concentration is associated with fructose metabolism, and in addition to increased fat mass, is known to mediate cardiorenal disease risk [58,59]. Consistent with this, we report an increase in systolic blood pressure associated with fructose administration. Unlike body weight, blood pressure was reported to increase in all studies included in the analysis, and the magnitude of the change was dependent on the duration of the study. The standardized mean difference for systolic blood pressure was two-fold higher in studies that lasted for longer than 12 weeks. Interestingly, in studies greater than 12 weeks, the subgroup analysis suggested an almost two-fold decrease in blood triglyceride concentration. Although there was no overall effect of study duration on body weight, this decrease could be linked to a change in body composition, specifically increased body fat mass or ectopic fat deposition.

Consumption of fructose is also associated with increased plasma glucose and insulin concentrations. Unlike glucose, the uptake and metabolism of fructose in the liver is virtually unregulated (see Regnault et al. [60] for review). As a result, excess fructose is rapidly converted into fatty acids and triglycerides through the induction of lipogenesis-promoting transcription factors sterol regulatory binding protein 1c (SREBP-1c) and carbohydrate response element binding protein (ChREBP) [61]. While insulin resistance is one of the main outcomes associated with long-term fructose feeding [62], short-term feeding has been shown to induce a transient insulin resistant state [9] that can significantly impact on insulin-mediated glucose metabolism, contributing to the development of insulin resistance and T2D. This transient insulin resistant state is believed to contribute to the loss of inhibition of gluconeogenic pathways [9], driving an increase in hepatic glucose output independent of pre- and postprandial blood glucose concentrations. The analysis presented herein suggests that the consumption of fructose, independent of duration, results in elevated blood glucose and insulin concentrations. This may contribute to the development of insulin resistance, and may eventually manifest as T2D.

As indicated in Figure 2, Figure 3, Figure 4, Figure 5 and Figure 6, there was a high degree of heterogeneity in all analyses conducted. We did not control for rodent strain in this analysis, which may account for the variability observed (Wistar-albino, 19 studies; Wistar-Kyoto, 1 study; Sprague-Dawley, 6 studies). To our knowledge, there have been no studies focused on rat strain-specific differences in fructose metabolism. Furthermore, two of the 26 studies used in this analysis reported fasting blood glucose, insulin and triglyceride concentrations collected after an overnight fast, which potentially contributed to the variability observed. This study was unable to control for the volume of beverage consumed per day, and it has been reported that rodents demonstrate increased intake when sugar is supplied in the form of a beverage when compared to chow [63]. Although a low concentration fructose beverage was provided to rodents in all studies included in this analysis, the volume consumed per day may have resulted in the daily caloric intake from fructose being higher than the beverage itself. However, this is consistent with human consumption of added sugars, as previously highlighted.

In conclusion, we observed that, independent of subtle study variations, consumption of a 10%–21% fructose beverage results in increased body weight in adult male rodents. This increase is accompanied by elevated systolic blood pressure and higher blood glucose, insulin and triglyceride concentrations. Furthermore, systolic blood pressure and blood triglyceride concentrations are sensitive to the duration of the fructose intervention, such that systolic blood pressure is elevated in long-term studies (over 12 weeks), while blood triglyceride concentrations are reduced. The physiological effects associated with low concentration fructose beverage consumption are consistent with development of the metabolic syndrome. Whilst we acknowledge that humans rarely consume fructose in isolation, we are consuming more fructose as either sucrose or HFCS than ever before. Understanding the metabolic effects of fructose, in isolation and when consumed with glucose, is critical to our understanding of metabolic disease. Rodent studies utilizing physiologically relevant fructose doses (~10% *w*/*w*), compared to those utilizing supraphysiological doses (~60% *w*/*w*), are important in allowing us to better elucidate the cellular mechanisms contributing to the development of type 2 diabetes, obesity and cardiovascular disease. This analysis confirms that administration of supraphysiological concentrations of fructose in rodent studies is not required to induce characteristics of the metabolic syndrome phenotype.

## Figures and Tables

**Table 1:** Characteristics and major outcome measures of studies included in the meta-analysis, including the effects of fructose beverage consumption on male body weight, systolic blood pressure and blood glucose, insulin and triglyceride concentrations.

Citation	Fructose Concentration (% *w*/*v*)	Duration of Intervention (Weeks)	*n* (% of Control)	Summary of Findings: Effect of Fructose Relative to Control				
				Body Weight	Systolic Blood Pressure	Blood Glucose	Blood Insulin	Blood Triglycerides
Ge et al. 2016 [23]	10	5	16 (50)	Significant increase	Data not reported	No effect	Significant increase	Significant increase
Prince et al. 2016 [24]	10	8	16 (50)	No effect	Data not reported	No effect	Data not reported	Significant increase
Ibrahim et al. 2015 [22]	10	20	14 (57.1) *	Significant increase (raw data not provided)	Data not reported	Significant increase	Significant increase	Significant increase
Litterio et al. 2015 [25]	10	8	18 (55.6)	No effect	Significant increase	Data not reported	Data not reported	Data not reported
Peredo et al. 2015 [26]	10	9	12 (50)	No effect	Significant increase	No effect	Significant increase	Significant increase
Al-Rasheed et al. 2014 [27]	10	8	20 (50)	Significant increase	Data not reported	Significant increase	Data not reported	Significant increase (raw data not provided)
Castro et al. 2014 [28]	10	3	40 (50)	No effect	Data not reported	No effect	Significant increase	Significant increase
Mahmoud and Elshazly, 2014 [29]	10	12	10 (50) *	Significant increase (raw data not provided)	Significant increase	Significant increase	Significant increase	Significant increase
Cardinali et al. 2013 [30]	10	8	16 (50)	Significant increase	Significant increase	Data not reported	Data not reported	Significant increase
Farina et al. 2013 [31]	10	3	20 (50) *	No effect	Data not reported	No effect	No effect	Significant increase
Larsen et al. 2013 [32]	10	26	12 (50)	No effect	Data not reported	No effect	No effect	No effect
Zarfeshani et al. 2012 [33]	21	10	12 (50)	No effect	Data not reported	No effect	Data not reported	No effect
Maiztegui et al. 2011 [34]	10	3	40 (50)	No effect	Data not reported	No effect	Significant increase	Significant increase
Shahraki et al. 2011 [35]	10	8	19 (47.4)	No effect (raw data not provided)	Data not reported	No effect	No effect	Significant increase
Francini et al. 2010 [50]	10	3	30 (50)	No effect	Data not reported	Significant increase	Significant increase	Significant increase
Giani et al. 2010 [36]	10	6	16 (50)	No effect	Significant increase	No effect	Significant increase	Data not reported
Atanasovska et al. 2009 [37]	10	12	28 (42.9)	No effect	Significant increase	Data not reported	Significant increase	Significant increase
Bi et al. 2009 [38]	10	32	30 (40)	Significant increase (raw data not provided)	Data not reported	No effect	Significant increase	Significant increase
Bi et al. 2008 [49]	10	38	21 (57.1)	Significant increase	Significant increase	No effect	Significant increase	Significant increase
Tan et al. 2008 [39]	10	32	21 (57.1)	Significant increase	Significant increase	No effect	Significant increase	Significant increase
Xing et al. 2008 [40]	10	34.7	21 (57.1)	Significant increase (raw data not provided)	Significant increase	No effect	Significant increase	Significant increase
Jalal et al. 2007 [41]	10	8	34 (29.4)	Significant increase	Data not reported	Significant increase	Data not reported	Significant increase
Sanchez-Lozada et al. 2007 [42]	10	8	14 (50)	No effect	Significant increase	No effect	Data not reported	No effect
Yadav et al. 2007 [43]	21	8	12 (50)	Significant increase	Data not reported	Significant increase	Significant increase	Significant increase
Xi et al. 2007 [44]	10	8	20 (50)	No effect	Significant increase	No effect	Significant increase	Significant increase
Shalam et al. 2006 [45]	10	2.86	12 (50)	Data not reported	Data not reported	Significant increase	Significant increase	Significant increase (raw data not provided)

* Where animal numbers per group were given as a range, the smallest number has been reported and used in the analysis.

**Table 2:** Summary statistics (mean (95% CI)) of fructose beverage concentration (% *w*/*v*), study duration and sample size (as a percentage of control) split for study duration (≤12 weeks or >12 weeks).

	Less Than or Equal to 12 Weeks (*n* = 20)	Greater Than 12 Weeks (*n* = 6)
**Fructose beverage concentration (% *w*/*v*)**	11.1 (9.5, 12.8)	10 *
**Study duration (weeks)**	7.0 (5.7, 8.4)	30.5 (23.7, 37.2)
**Sample size (% of control)**	48.8 (46.4, 51.1)	53.1 (45.7, 60.4)

* Fructose concentration was the same in all studies (10% *w*/*v*).
